# Anti-MrkA Monoclonal Antibodies Reveal Distinct Structural and Antigenic Features of MrkA

**DOI:** 10.1371/journal.pone.0170529

**Published:** 2017-01-20

**Authors:** Qun Wang, Yan Chen, Romana Cvitkovic, Meghan E. Pennini, Chew shun Chang, Mark Pelletier, Jessica Bonnell, Adem C. Koksal, Herren Wu, William F. Dall’Acqua, C. Kendall Stover, Xiaodong Xiao

**Affiliations:** 1 Dept. of Infectious Disease and Vaccines, MedImmune, Gaithersburg, MD, United States of America; 2 Dept. of Antibody Discovery and Protein Engineering, MedImmune, Gaithersburg, MD, United States of America; CIP, NCI-Frederick, NIH, UNITED STATES

## Abstract

Antibody therapy against antibiotics resistant *Klebsiella pneumoniae* infections represents a promising strategy, the success of which depends critically on the ability to identify appropriate antibody targets. Using a target-agnostic strategy, we recently discovered MrkA as a potential antibody target and vaccine antigen. Interestingly, the anti-MrkA monoclonal antibodies isolated through phage display and hybridoma platforms all recognize an overlapping epitope, which opens up important questions including whether monoclonal antibodies targeting different MrkA epitopes can be generated and if they possess different protective profiles. In this study we generated four anti-MrkA antibodies targeting different epitopes through phage library panning against recombinant MrkA protein. These anti-MrkA antibodies elicited strong *in vitro* and *in vivo* protections against a multi-drug resistant *Klebsiella pneumoniae* strain. Furthermore, mutational and epitope analysis suggest that the two cysteine residues may play essential roles in maintaining a MrkA structure that is highly compacted and exposes limited antibody binding/neutralizing epitopes. These results suggest the need for further in-depth understandings of the structure of MrkA, the role of MrkA in the pathogenesis of *Klebsiella pneumoniae* and the protective mechanism adopted by anti-MrkA antibodies to fully explore the potential of MrkA as an efficient therapeutic target and vaccine antigen.

## Introduction

*Klebsiella pneumoniae (KP)* is an etiological agent for many serious nosocomial infections[1]. The increased incidences of infections caused by expanded spectrum antibiotics resistant *KP* strains coupled with dwindling antibiotics pipelines pose significant health challenges [2]. To combat such infections alternative strategies are being pursued in addition to identifying new antibiotics. Monoclonal antibodies represent a promising new class of anti-*KP* therapeutics as they employ a different protective mechanism and use different targets than those of antibiotics. However, the exquisite target specificity of antibodies often limits their usefulness due to their restricted coverage of only a small percentage of prevalent *KP* strains. For example, capsule and LPS are validated protective antigens [3–5]. There are more than 77 different capsule and 9 LPS serotypes[6, 7], necessitating the inclusion of multiple components in a vaccine regimen and making the generation of antibodies, antibody combinations, and the formulation of the antibody combinations very challenging [8]. Thus, identification of protective antibody antigens shared by different *KP* strains is highly desirable for adopting an antibody based anti-*KP* strategy. A similar strategy was successfully used against *Pseudomonas* infection [9]. Despite extensive efforts using various approaches very few such targets have been discovered against *KP*. One such target, PNAG, was reported to be present on the surface of a wide collection of bacterial and fungal strains including *KP*, with anti-PNAG antibody F598 currently under clinical development [10]. Its impact on *KP* infection remains to be seen.

We reported in a previous study the identification of MrkA as a common protein antigen expressed by the majority of *KP* strains [11]. MrkA is a major component of the type III fimbria complex. It is involved in biofilm formation and establishment of infection [12–14]. Its amino acid sequence is highly conserved among the majority of enterobactereace strains analyzed. Using a target-agnostic approach we found that anti-MrkA antibodies generated from both hybridoma and phage display platforms displayed potent *in vitro* opsonophagocytic (OPK) activity, biofilm formation inhibitory activity and protective *in vivo* activities in reducing organ burden and extending survival after challenges with *KP*. It is expressed by more than 60% of clinical *KP* strains when screened against one anti-MrkA monoclonal antibody KP3 [11]. Interestingly, despite the apparently significant differences between the two antibody discovery platforms all the antibodies we identified targeted an overlapping epitope. It is important to understand if antibodies targeting different epitopes can be identified and demonstrate different protective profiles[15].

To address these questions we made a concentrated effort to select anti-MrkA antibodies targeting different epitopes using phage display platform. We found that antibodies targeting different epitopes can be identified by panning naive human single-chain variable fragment (scFv) antibody phage libraries against purified recombinant MrkA protein. However, epitopes of all anti-MrkA mAbs fall within a narrowly restricted range. These antibodies displayed *in vitro* and *in vivo* activities that are comparable to the previously identified and characterized anti-MrkA antibody KP3. The combination of different antibodies did not have any additional benefit. These findings suggest that MrkA may play a complex role in *KP* pathogenesis and a better understanding of the mechanism may help to utilize anti-MrkA antibodies more efficiently as therapeutics and MrkA as a vaccine antigen.

## Materials and Methods

### *Klebsiella pneumoniae* strains and animal models

All *KP* strains and animal models used were as described in a previous study [11]. All animal experiments were conducted in compliance with the Animal Welfare Act and the Guide for the Care and Use of Laboratory Animals, National Research Council, 1996, in an AAALAC-accredited rodent facility within MedImmune in accordance with approved IACUC protocol (MI-15-0026). After *KP* challenges animals were monitored twice daily for *KP* infection-induced adverse effects including labored breathing, hunched posture, piloerection, and immobility, etc. for up to seven days. Upon the completion of the studies, surviving animals were euthanized by CO2 inhalation per GUID-0065.

### Recombinant MrkA Protein preparation

His-tagged recombinant MrkA was expressed and purified as described previously for large scale production with modifications [11]. MrkA expressed in the *E*.*coli* host strain BL21(DE3) stayed mostly in the inclusion body. Buffer containing eight molar urea was used to solubilize MrkA, and the his-tagged MrkA was purified using HisTrap HP column (GE Healthcare) as described with the exception that denatured MrkA was loaded directly to the affinity column and purified under the denaturing condition without refolding first. Monomeric and oligomeric MrkA were eluted together without further separations. Eluted MrkA fractions were collected and dialyzed against PBS buffer and ready for biotin labeling and panning. For biotin-labeling, the labeling kit from Pierce was used and the manufacturer’s protocol was followed.

### Panning and screening

Panning selection was performed in a solution format using a Kingfisher automated system as described with modifications [16]. Naïve scFv phage display libraries used in this study were described previously [17]. Panning antigen MrkA was biotinylated and 0.3 μg was used in each of the first two rounds of panning. For selections that needed a third round, biotinylated MrkA was reduced to 0.1 μg. When the phage output was improved to more than 100 folds compared to that of the first round, panning selection was stopped and high throughput screenings were initiated.

The first round of screening was based on specific bindings to MrkA. scFv.Fc expressed through the pSplice.V5 vector in E.coli strain Top 10 (Invitrogen) was used in a homogeneous time resolved FRET (HTRF) based assay to screen for specific binders [18, 19]. Resulted MrkA specific binders were consolidated, sequenced, and the unique clones were used to prepare plasmids for mammalian cell transfection, scFv.Fc expression, and OPK analysis as described previously [18]. Those scFv.Fc clones showing positive OPK activities were binned based on a bio-layer interferometry (BLI) assay. Representative clone from each bin was converted to human IgG1 format for in-depth functional characterizations and MrkA mutational analysis.

### Bio-layer interferometry (BLI) for affinity measurement and epitope binning

Clones that were positive in the binding and OPK assays were used in bio-layer interferometry based assay to assess their apparent affinities and relative binding epitopes.

For affinity measurement, two different formats were used. The first was to use an IgG against a mixture of monomeric and oligomeric MrkA. The second was to use a Fab against a monomeric MrkA. A ForteBio Octet QK384 instrument was used to study kinetics of the anti-MrkA mAbs. All the assays were done at 200 μl/well in ForteBio 10x kinetic buffer at 30°C. 0.3 μg/ml of biotinylated-MrkA was loaded on the surface of streptavidin biosensors (SA) for 400 s reaching levels between 1.0 and 1.5 nm followed by a 300 s biosensor washing step. Association of MrkA on the biosensor to the individual mAbs in solution (0.274–200 nM) was analyzed for 600 s. Dissociation of the interaction was probed for 600 s. Any systematic baseline drift was corrected by subtracting the shift recorded for a sensor loaded with ligand but incubated without analyte. Octet Data Analysis software version 8.0 was used for curve fitting with the binding equations available for a 1:1 interaction model. Global analyses were done using nonlinear least squares fitting. Goodness of fit for the data were assessed by the generated residual plots, R2 and χ2 values.

Epitope binning was done on a ForteBio Octet QK384. Biotinylated-MrkA was captured onto streptavidin biosensors and coated with testing mAbs (antibody bin) at a saturating concentration of 200 nM for 600 seconds. The epitopes of other mAbs were probed in relation to testing mAbs by assaying the testing mAb-coated biosensors in 100 nM each of the other mAbs together with equal concentration of the testing mAb. All graphs were overlaid and aligned at the baseline.

### Peptide array analysis

An array of 12 peptides covering the full length mature MrkA protein from *Klebsiella pneumoniae* strain MGH78578 was synthesized(New England Peptide) excluding the signal peptide [20]. Each peptide has 20 amino acid residues, and except for the first and the last peptides each has five amino acid overlapping with flanking peptides. Each peptide also has biotin at its N–terminus. The peptide array is used to explore the binding epitopes of the anti-MrkA mabs by ELISA as described with modifications [21]. Briefly, an ELISA plate was coated with 5 μg/ml of neutravidin (Pierce) at 4°C overnight. The plate was blocked with PBST (PBS + 0.1% Tween) supplemented with 4% BSA at room temperature for one hour. The plate was then incubated with 50 μg /ml of MrkA peptides at room temperature for one hour. Five μg/ml of the anti-MrkA antibodies were then added to the peptide array and incubated for one hour. The plate was washed and goat anti-huIgG Fcγ-HRP (for phage clones) or anti-mIgG Fcγ-HRP (for hybridomas) (Jackson ImmunoResearch) were added. The plate was washed and TMB substrate (KPL, Gaithersburg, MD) and 0.1 N HCL stop buffers added. OD450 was measured in an Envision Multilabel plate reader (Perkin Elmer).

### Flow cytometry analysis of antibody binding to *Klebsiella pneumoniae*

Bacteria was cultured in 2xYT broth overnight and then diluted into FACS buffer (PBS with 0.5% of Bovine Serum Albumin) to an approximate concentration of 2x10^7^ CFU/ml. Bacteria (1x10^6^) were incubated with anti-MrkA antibodies or with negative control antibody for 1 hour at 4°C with gentle shaking in 96 well round bottom plates. Plates were washed with FACS buffer and centrifuged (3500rpm, 5min), followed by incubation with Alexa Fluor 647 goat anti-human IgG secondary antibody (Life Technologies). Plates were incubated in the dark for 1 hour at 4°C with gentle shaking and washed twice with FACS buffer. Samples were measured in a BD LSR II (BD Biosciences) and analyzed using the FlowJo program.

### Opsonophagocytic killing (OPK) *in vitro* activity assessment

Representative clones from each binning group including clones 1, 4, 5, and 6 were converted to IgG1, expressed, purified and analyzed in OPK assay. Briefly, log phase culture of luminescent *KP* strains (Lux) was diluted to ~ 2x10^6^ cells/ml. Bacteria, diluted baby rabbit serum providing complement (Cedarlane, 1:10), dimethylformamide (DMF) differentiated HL-60 cells or freshly isolated polymorphonuclear leukocytes (PMN) cells, and anti-MrkA IgGs were mixed in 96-well plates and incubated at 37°C for two hours with shaking (250 rpm). The relative light units (RLUs) were then measured using an Envision Multilabel plate reader (Perkin Elmer). The percentage of killing was determined by comparing RLU derived from assays with no antibodies to RLU obtained from anti-*KP* mAbs and a negative control mAb.

### *Klebsiella pneumoniae in vivo* challenge studies

For evaluation of the *in vivo* protective activities of the newly identified antibodies we used a previously established acute pneumonia models[11]. C57BL/6 mice were inoculated with 1-2x10^8^ CFU of a multi-drug resistant isolate intranasally. KP3, a human IgG1 control antibody R347, and the newly identified anti-MrkA antibodies were given via intraperitoneal (IP) route either 24 hour prior to bacterial challenge for prophylaxis or one hour post bacterial challenge for therapy. Mouse survival was monitored daily for a minimum of five days until up to day 8. Survival data of representative experiment was plotted in Prism. In addition antibodies in various combinations were tested in the therapeutic model to see if synergistic effect could be achieved.

## Results

### Panning summary

**Fig 1:**
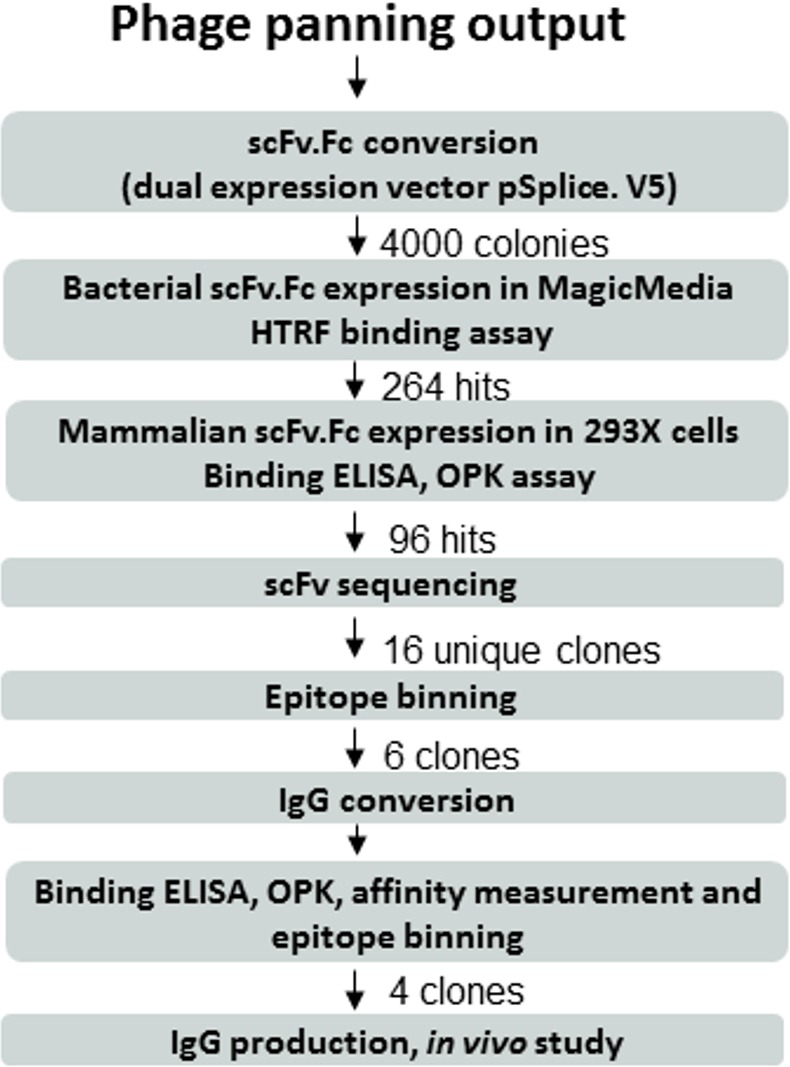
Phage panning output screening cascade. More than 4000 colonies were picked for high throughput screening after phage panning, scFv.Fc conversion and transformation. Four clones including clones 1, 4, 5, and 6 were selected for further characterization.

For panning studies monomeric MrkA was not separated from oligomeric MrkA as we have shown in our previous study that both forms induced similar levels of protection when used as vaccine antigens [11]. Furthermore it was not clear which format would present protective epitopes better during the *in vitro* antibody selection process. After the second or third round of selection the panning output was improved to more than 100 fold compared to that of the first round. The panning output was converted to scFv.Fc in pSplice.V5 and subjected to high throughput screening [18]. The screening process is summarized in Fig 1. Starting with more than 4000 picked bacterial colonies we succeeded in isolating four different MrkA specific, OPK positive antibodies representing different binding epitopes. These four antibodies were converted to the human IgG1 format and subjected to further characterization. They were named anti-MrkA clones 1, 4, 5, and 6.

### Antibody characterizations

**Fig 2:**
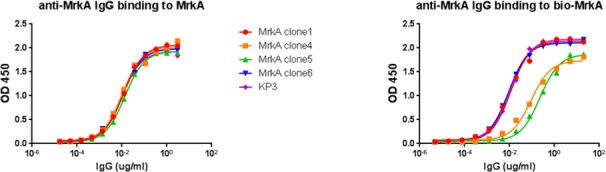
Anti-MrkA antibody binding is influenced by the antigen presentation format. MrkA protein, either coated directly to the ELISA plate (left panel), or captured by streptavidin after biotinylation (right panel), was recognized differently by the anti-MrkA antibodies.

The four clones 1, 4, 5, and 6 were expressed as human IgG1 in 293 free style cells (Invitrogen) and purified. While they maintained robust binding activities, it is apparent that the ELISA format impacted binding significantly. There was no difference in binding when recombinant MrkA was coated directly onto the ELISA plate representing an aggregated or multimeric form (Fig 2 left panel), whereas clone 4 and 5 displayed weakened binding when biotinylated MrkA was captured onto the neutravidin coated ELISA plate suggesting their preference for the multimeric format (Fig 2 right panel). The apparent affinities were measured between 3–10 nM (Table 1) by a bio-layer interferometry (BLI) method in an IgG format.

**Table 1:** K_D_ measurement in IgG format against a mixture of monomeric and multimeric MrkA.

IgG	K_D_	K_on_ (x 10^4^ 1/Ms)	K_off_ (x 10^−4^ 1/s)	R^2^
clone 1	3.25 nM	5.3	1.7	0.989
clone 4	3.61 nM	4.06	1.46	0.985
clone 5	3.54 nM	2.6	9.2	0.996
clone 6	8.80 nM	2.2	2.0	0.996
KP3	0.15 nM	7.6	1.2	0.993

**Fig 3:**
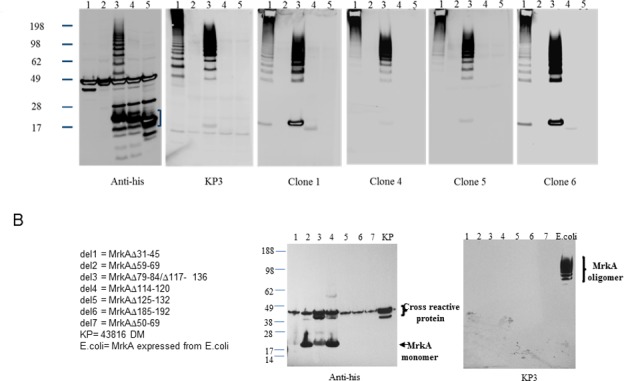
Mutational analysis of MrkA and mAb characterizations by Western blot. 3A. Western blot against full length MrkA and deletion mutants. Protein samples were resolved on a 4–12% SDS-PAGE gel under reducing condition and subjected to Western blot analysis by a murine anti-his mAb, KP3, and clones 1–6 IgGs as indicated underneath each blot. Sample arrangement is as follow: Lane 1, cell lysate from KP strain 43816DM; Lane 2, E.coli BL21 strain control; Lane 3–5 BL21 strain expressing his-tagged recombinant MrkA, MrkA with N terminal 40 amino acid deletion, and MrkA with C terminal 32 amino acid deletion, respectively. In the anti-his blot, a bracket indicates the positions of the monomeric, full length MrkA as well as the deletion mutants. 3B. Western blot analysis of various MrkA deletion mutants. MrkA deletion mutants 1 to 7 (del1-7) as indicated on the left were expressed in BL21 strain, resolved on a 4–12% SDS-PAGE gel under reducing condition, and subjected to Western blot detection by anti-his and KP3 antibodies as indicated. Lane arrangement: KP is the lysate prepared from 43816DM strain and E.coli represents lysate prepared from BL21 expressing the recombinant, his-tagged MrkA. 1–7 represent the mutants with corresponding numbers (del 1–7) described on the left. For both 3A and B, numbers to the left of the gel indicate the molecular weight in kDa.

Western blot data showed that all clones recognized the multimeric MrkA, either from *KP* extract or recombinant expression in *E*.*coli* predominantly (Fig 3A). Clones 1 and 6 also recognized the monomeric MrkA from both endogenous and recombinant sources to certain extent in agreement with the ELISA data suggesting the preference of multimeric form of MrkA by clones 4 and 5 and to a lesser extent by 1 and 6 (Fig 2). When monomeric MrkA was used in a BLI assay against the Fab format of the four clones it is surprising to find that only clones 1 and 5 retained binding activities to different levels whereas clone 4 and KP3 lost the binding entirely (Table 2). MrkA is especially intolerant to mutations and sub-clone and expression of fragments from MrkA often resulted in loss of multimeric format or no expression completely. Deletions of the N terminal 40 amino acids or the C terminal 32 amino acids resulted in the inability to form the multimeric form (Fig 3A, anti-his panel). This is in agreement with a previous study suggesting the involvement of both N and C termini in the oligomer formation by a similar protein [22]. Similarly small deletions within the MrkA also resulted in the inability of MrkA to form the multimeric form or no expression (Fig 3B and data not shown).

**Table 2:** K_D_ measurement in Fab format against monomeric MrkA. ND, not detectable; N/A, not applicable.

Fab	K_D_	K_on_ (x 10^6^ 1/Ms)	K_off_ (x 10^−3^ 1/s)	R^2^
Clone 1	2.76 nM	0.15	0.34	0.998
Clone 4	ND	ND	N/A	--
Clone 5	1520 nM	0.05	78.2	0.997
KP3	ND	ND	N/A	--

**Fig 4:**
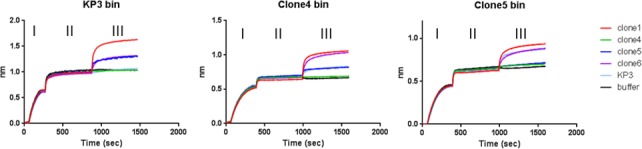
Epitope analysis by BLI. Epitope binning was performed against three test article including KP3, clone 4 and 5 as described in the materials and method. There are three phases (I, II, and III) for each graph. In phase I biotin-labeled MrkA protein was loaded to the neutravidin probe. In phase II one of the mAbs (test articles) as indicated above each graph bound to the immobilized MrkA. In phase III different mAbs as indicated were mixed with the test article mAb and loaded to the probe.

The fact that all the anti-MrkA mAbs prefer the multimeric form determined that mutational analysis was not an appropriate method for epitope analysis. Instead, we used a BLI based approach to study the relative positions of the epitopes of the mAbs. In binning experiments, IgG clone 1 appears to possess an epitope that is different from all others, whereas IgGs clone 4, 5, 6, and the clone identified from a previous campaign, KP3, showed epitopes that overlap to a limited extent as revealed by different binning set-up (Fig 4). Clone 4 binding to MrkA was completely blocked in the BLI assay by all other antibodies but clone 4 failed to block the binding by others, suggesting that clone 4 possesses very unique epitope features (Fig 4).

**Fig 5:**
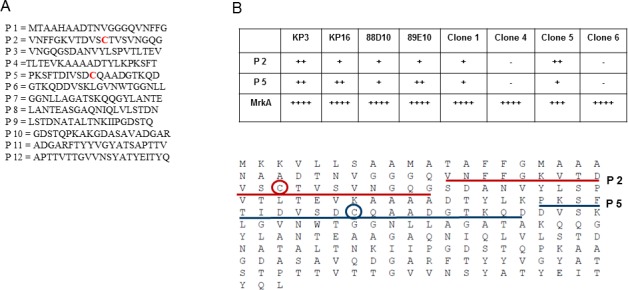
Peptide array analysis. 5A. The twelve peptides included in the peptide array (P 1-P 12): 5B. Upper panel: summary of the array analysis. Only the peptides showing any sign of binding activity by the anti-MrkA mAbs were included. “++++” indicates an ELISA signal 10 fold above background, “+++” 5 fold above background, “++” 2 fold above background and “+” marginally above background. KP3, KP16, 88D10, and 89E10 are mAbs identified from a previous study [11]. Lower panel: The two peptides (P 2 and P 5) showing positive binding are underlined in the context of MrkA. The two cysteines are circled.

In parallel, we designed a peptide array with twelve 20-mers covering the full length of the mature MrkA (Fig 5A). We failed to identify a single peptide that can account for the full binding activity by any of the antibodies against the full length recombinant MrkA. Instead we observed consistently that peptide 2 and 5 displayed weak bindings by the majority of the antibodies we have identified. No other peptide showed any activity (Fig 5B upper panel). Interestingly both peptide 2 and 5 carry a cysteine residue (Fig 5B lower panel). Crystal structure of a similar protein FimH from *E*.*coli* showed the disulfide bond formation between the two cysteines in the similar positions[22]. Furthermore, when we mutated either of the cysteine to alanine we did not detect any expression (data not shown) consistent with a previous study with *KP* MrkA[20]. In the absence of a crystal structure, our data provide the best indication that MrkA adopts a similar compact structure to FimH with the disulfide bond playing an essential role[22]. Residues surrounding the two cysteines as represented by peptides 2 and 5 are likely brought to close proximity and make up the discontinuous epitopes for the antibodies we have isolated. It is surprising to find that other regions represented by the rest of the peptides failed to elicit any antibody despite all the efforts, suggesting a very restricted accessibility.

### OPK activity is important for *in vivo* protection

**Fig 6:**
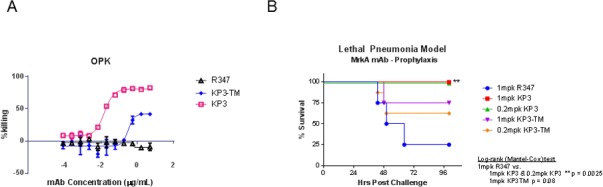
OPK activity is important for in vivo protective activity. KP3-TM mutation was generated and tested in both in vitro OPK (4A) and in vivo challenge assay (4B). Significant reduction was seen in the OPK assay and a trend towards significance was seen in the in vivo challenge assay. R347 is a human IgG_1_ isotype control.

In order to understand the roles of OPK activity for *in vivo* protection, we generated KP3 IgG with TM mutations in the Fc fragment to eliminate its effector functions [23]. The OPK activity was reduced significantly (Fig 6A) and the reduction in OPK activity corresponded to a reduction in protection in an *in vivo* challenge model (Fig 6B). However, neither the OPK activity nor the *in vivo* protection was completely eliminated. These data suggest that OPK is important to the protective mechanism of the anti-MrkA antibody KP3.

### Antibody binding to live bacteria as examined by flow cytometry

**Fig 7:**
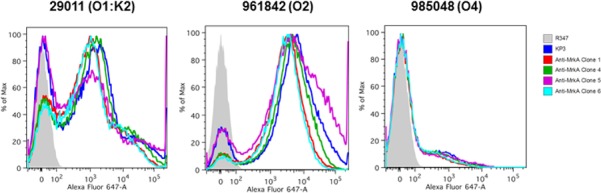
Serotype-independent binding to KP strains by the new antibodies. Flow cytometry experiment was used to gauge the binding of the four new antibodies against three WT KP strains of different serotype as indicated. KP3 serves as the positive control and R347 is a human IgG_1_ isotype control.

To determine whether the antibodies bind to *KP*, flow cytometry analysis was performed against live bacteria of different serotypes. We found that all four new antibodies recognized the three isolates tested. Even though each antibody bound to different isolates to different extent, there were no significant differences among the antibodies (Fig 7). Furthermore, selected isolates were inoculated by intranasal route, and bronchalveolar lavage was collected three hours post infection. We then analyzed the anti-MrkA antibody binding to these *in vivo* passaged bacteria. We confirmed that anti-MrkA mAbs bound to the *in vivo* grown bacteria in the same fashion as the *in vitro* culture grown bacteria (data not shown).

### Antibody characterization by OPK assay

**Fig 8:**
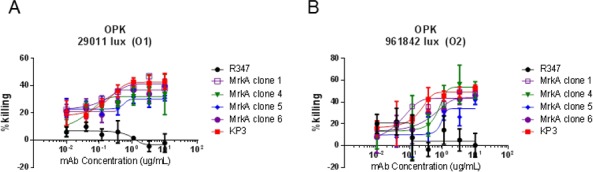
Serotype independent OPK activity by the new antibodies. Two KP strains with LPS serotypes O1 and O2 respectively were used in the OPK assay. All newly isolated antibodies displayed comparable OPK activities to that of KP3. R347 is a human IgG_1_ isotype control.

Clones 1, 4, 5, and 6 were selected for further analysis due to their different epitopes and positive OPK activity in the scFv-Fc format during the screening process. We performed OPK analysis with their IgG1 counterparts, and they all displayed positive OPK activity comparable to that of KP3 against *KP* of different serotypes (Fig 8).

### Antibody protective effects in an *in vivo* challenge model

**Fig 9:**
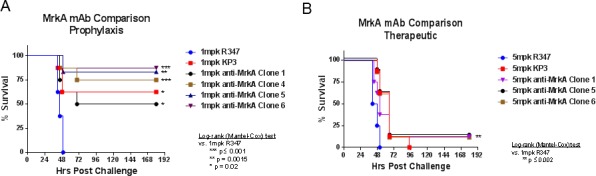
In vivo protection activity. The newly identified antibodies were tested in in vivo challenge models under both prophylaxis and therapeutic settings. In the prophylaxis setting antibodies were given 24 hours prior to KP challenge (7A), whereas in the therapeutic setting antibodies were given one hour after KP challenge (7B). There were eight animals in each group. R347 is a human IgG_1_ isotype control.

Reflecting their comparable bacterial binding and OPK activity, all newly identified antibodies displayed similarly potent *in vivo* protective activity in a prophylaxis model (Fig 9A). At 1 mg/kg dose all antibodies conferred near complete protection. However, in a therapeutic model, only modest protection was seen at a dose as high as 5 mg/kg (Fig 9B). There did not seem to be significant differences between antibodies targeting different epitopes in their protective activities in either model.

### Combinations of antibodies did not show additional protective effect

**Fig 10:**
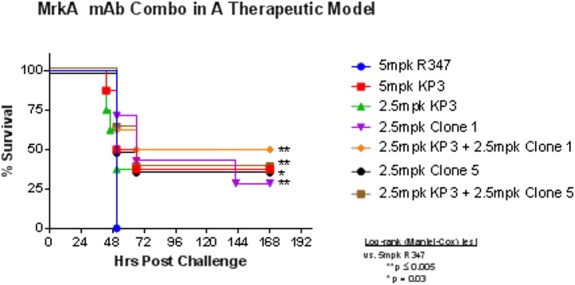
No synergistic effect by antibody combinations in the therapeutic model. KP3 was combined with either clone 1 or 5 in equal amount as indicated and tested in a therapeutic model. No significant improvement was observed. There were eight animals in each group. R347 is a human IgG_1_ isotype control.

Antibody combinations in the anti-bacterial field have achieved some very promising results. Thus combinations of the anti-MrkA antibodies were investigated. We failed to observe significant additive or synergistic effects when we combined KP3 with either of the two newly identified antibodies clones 1 and 5 (Fig 10). More complex combinations with up to three mAbs also failed to show any additional benefit (data not shown).

## Discussions

In an effort to develop an antibody based therapy that is broadly applicable to treatment of infections caused by *KP* strains of different serotypes, we identified MrkA as a potential target through an agnostic approach. MrkA is an appealing target due to its broad strain coverage and we have shown that anti-MrkA antibodies possess protective activities *in vivo*. One interesting observation during the agnostic identification of the protective antibodies was that they all targeted an overlapping epitope. It has been well established that epitopes play important roles in determining the various biological functions of monoclonal antibodies. Additionally, it has been demonstrated in different therapeutic areas that combination of antibodies possessing different epitopes can have potent biological activities that are not possessed by any of the individual monoclonal antibodies.

In this study we attempted to understand if epitopes are a critical factor in deciding anti-MrkA antibody protective effects by identifying and comparing antibodies targeting non-overlapping or minimally overlapping epitopes on MrkA. In addition, we tested whether a combination of antibodies directed against different epitopes could contribute to better protection than that conferred by a single monoclonal antibody.

Several phage clones have been identified in this study based on their positive OPK activities and non- or minimally overlapping epitopes either with KP3 or among themselves. Despite the different apparent binding affinities and epitopes, they displayed similar *in vitro* OPK and *in vivo* protective activities in the models we have tested. It is not entirely clear what additional factors are deemed most critical in determining protective effects. Furthermore, clone 1 and 6 recognize the monomeric MrkA, which has not been detected as a dominant form in nature. This unique biochemical feature failed to correlate significantly with any biological functions.

Unlike what has been elegantly described for anti-Botulinum toxin[24], against which the combination of three different monoclonal antibodies targeting different epitopes displayed potent protective activities, we failed to observe any beneficial effect by combining two or three anti-MrkA antibodies. This is likely due to the different protective mechanisms used by the antibodies against the two targets. Antibodies against botulinum protect the host through an increase in functional affinities and/or better inhibition of toxin interactions with the cell surface. Synergistic activities have been observed with oligoclonal antibodies against other targets including tetanus toxin and HIV[25, 26]. In addition, such activities were also reported for oligoclonal antibodies against a target with autoimmune disease indications[27]. It has not been established that it is the same case with OPK activity. From our current study there appears to be a lack of such a correlation. There have been reports that antibodies targeting different antigens on the same pathogen can benefit from combinations[28]. It is likely that the outcomes of combinations are antigen dependent. Additionally we observed that dose response did not always hold true for all the anti-MrkA antibodies in *in vivo* protection models and this adds another layer of complexity as the optimum doses to be used in antibody combinations for each of the mAbs are not straightforward. Finally, our results showed that the epitopes of all the anti-MrkA mAbs are in close proximity even if they are different such as clone 1. This combined with the multimeric feature of MrkA may contribute to reduce some of the beneficial effect brought about by combining antibodies targeting drastically different epitopes as seen with anti-botulinum antibodies. Clearly, more research is required to understand if and how OPK activity-based antibodies can benefit from such a combination targeting antigens of different nature.

Another intriguing observation is that there was a lack of direct correlation between anti-MrkA antibody binding intensity to the bacteria and their *in vivo* protective effect. Although both *in vitro* and *ex vivo* binding displayed similar patterns by our anti-MrkA mAbs, we cannot rule out the possibility that at unknown stages of infection the binding could be different among different isolates. We did observe in flow cytometry experiment that despite the positive bindings by anti-MrkA antibodies to a wide collection of *KP* isolates, there were various percentages of bacteria that were negative in binding within each population. It would be interesting to study these subpopulations and understand if they constitute anti-MrkA antibody resistant mechanism. All these require further investigation of the roles of MrkA in the process of infection establishment, pathogenesis, and expression dynamics. There has been a lack of anti-MrkA mAbs until recently when we generated a collection of mAbs targeting different epitopes of MrkA. These should be powerful tools for in depth studies of *KP* pathogenesis and helping to develop the most potent anti-KP antibodies. Meanwhile these antibodies should help to delineate the very interesting and complex structural features of MrkA.

## References

[1] Broberg CA, Palacios M, Miller VL. Klebsiella: a long way to go towards understanding this enigmatic jet-setter. F1000Prime Rep. 2014;6:64 Epub 2014/08/29. 10.12703/P6-64 25165563 PMC4126530

[2] McKenna M. Antibiotic resistance: the last resort. Nature. 2013;499(7459):394–6. Epub 2013/07/28. 10.1038/499394a 23887414

[3] Clements A, Jenney AW, Farn JL, Brown LE, Deliyannis G, Hartland EL, et al Targeting subcapsular antigens for prevention of Klebsiella pneumoniae infections. Vaccine. 2008;26(44):5649–53. Epub 2008/08/30. 10.1016/j.vaccine.2008.07.100 18725260

[4] Cross AS. Anti-endotoxin vaccines: back to the future. Virulence. 2014;5(1):219–25. PubMed Central PMCID: PMC3916378. 10.4161/viru.25965 23974910 PMC3916378

[5] Edelman R, Taylor DN, Wasserman SS, McClain JB, Cross AS, Sadoff JC, et al Phase 1 trial of a 24-valent Klebsiella capsular polysaccharide vaccine and an eight-valent Pseudomonas O-polysaccharide conjugate vaccine administered simultaneously. Vaccine. 1994;12(14):1288–94. 7856293 10.1016/s0264-410x(94)80054-4

[6] Brisse S, Passet V, Haugaard AB, Babosan A, Kassis-Chikhani N, Struve C, et al wzi Gene sequencing, a rapid method for determination of capsular type for Klebsiella strains. Journal of clinical microbiology. 2013;51(12):4073–8. PubMed Central PMCID: PMC3838100. 10.1128/JCM.01924-13 24088853 PMC3838100

[7] Hansen DS, Mestre F, Alberti S, Hernandez-Alles S, Alvarez D, Domenech-Sanchez A, et al Klebsiella pneumoniae lipopolysaccharide O typing: revision of prototype strains and O-group distribution among clinical isolates from different sources and countries. Journal of clinical microbiology. 1999;37(1):56–62. PubMed Central PMCID: PMC84167. 9854064 10.1128/jcm.37.1.56-62.1999 PMC84167

[8] Donta ST, Peduzzi P, Cross AS, Sadoff J, Haakenson C, Cryz SJ Jr., et al Immunoprophylaxis against klebsiella and pseudomonas aeruginosa infections. The Federal Hyperimmune Immunoglobulin Trial Study Group. J Infect Dis. 1996;174(3):537–43. Epub 1996/09/01. 8769611 10.1093/infdis/174.3.537

[9] DiGiandomenico A, Warrener P, Hamilton M, Guillard S, Ravn P, Minter R, et al Identification of broadly protective human antibodies to Pseudomonas aeruginosa exopolysaccharide Psl by phenotypic screening. J Exp Med. 2012;209(7):1273–87. Epub 2012/06/27. 10.1084/jem.20120033 22734046 PMC3405507

[10] Cywes-Bentley C, Skurnik D, Zaidi T, Roux D, Deoliveira RB, Garrett WS, et al Antibody to a conserved antigenic target is protective against diverse prokaryotic and eukaryotic pathogens. Proceedings of the National Academy of Sciences of the United States of America. 2013;110(24):E2209–18. PubMed Central PMCID: PMC3683766. 10.1073/pnas.1303573110 23716675 PMC3683766

[11] Wang Q, Chang CS, Pennini M, Pelletier M, Rajan S, Zha J, et al Target-Agnostic Identification of Functional Monoclonal Antibodies Against Klebsiella pneumoniae Multimeric MrkA Fimbrial Subunit. J Infect Dis. 2016;213(11):1800–8. 10.1093/infdis/jiw021 26768253

[12] Jagnow J, Clegg S. Klebsiella pneumoniae MrkD-mediated biofilm formation on extracellular matrix- and collagen-coated surfaces. Microbiology. 2003;149(Pt 9):2397–405. 10.1099/mic.0.26434-0 12949165

[13] Langstraat J, Bohse M, Clegg S. Type 3 fimbrial shaft (MrkA) of Klebsiella pneumoniae, but not the fimbrial adhesin (MrkD), facilitates biofilm formation. Infect Immun. 2001;69(9):5805–12. Epub 2001/08/14. 10.1128/IAI.69.9.5805-5812.2001 11500458 PMC98698

[14] Murphy CN, Clegg S. Klebsiella pneumoniae and type 3 fimbriae: nosocomial infection, regulation and biofilm formation. Future Microbiol. 2012;7(8):991–1002. Epub 2012/08/24. 10.2217/fmb.12.74 22913357

[15] Xiao X, Wu H, Dall'Acqua WF. Immunotherapies against antibiotics-resistant Klebsiella pneumoniae. Hum Vaccin Immunother. 2016:0. 10.1080/21645515.2016.1210746 PMC5215587 27431874

[16] Lillo AM, Ayriss JE, Shou Y, Graves SW, Bradbury AR, Pavlik P. Development of phage-based single chain Fv antibody reagents for detection of Yersinia pestis. PloS one. 2011;6(12):e27756 PubMed Central PMCID: PMC3234238. 10.1371/journal.pone.0027756 22174746 PMC3234238

[17] Vaughan TJ, Williams AJ, Pritchard K, Osbourn JK, Pope AR, Earnshaw JC, et al Human antibodies with sub-nanomolar affinities isolated from a large non-immunized phage display library. Nature biotechnology. 1996;14(3):309–14. 10.1038/nbt0396-309 9630891

[18] Xiao X, Chen Y, Mugabe S, Gao C, Tkaczyk C, Mazor Y, et al A Novel Dual Expression Platform for High Throughput Functional Screening of Phage Libraries in Product like Format. PloS one. 2015;10(10):e0140691 PubMed Central PMCID: PMC4607404. 10.1371/journal.pone.0140691 26468955 PMC4607404

[19] Newton P, O'Shea D, Wells E, Moakes K, Dunmore R, Butler RJ, et al Development of a homogeneous high-throughput screening assay for biological inhibitors of human rhinovirus infection. Journal of biomolecular screening. 2013;18(3):237–46. 10.1177/1087057112469047 23207740

[20] Chan CH, Chen FJ, Huang YJ, Chen SY, Liu KL, Wang ZC, et al Identification of protein domains on major pilin MrkA that affects the mechanical properties of Klebsiella pneumoniae type 3 fimbriae. Langmuir. 2012;28(19):7428–35. Epub 2012/04/25. 10.1021/la300224w 22524463

[21] Xiao X, Chen Y, Varkey R, Kallewaard N, Koksal AC, Zhu Q, et al A novel antibody discovery platform identifies anti-influenza A broadly neutralizing antibodies from human memory B cells. mAbs. 2016;8(5):916–27. PubMed Central PMCID: PMC4968088. 10.1080/19420862.2016.1170263 27049174 PMC4968088

[22] Choudhury D, Thompson A, Stojanoff V, Langermann S, Pinkner J, Hultgren SJ, et al X-ray structure of the FimC-FimH chaperone-adhesin complex from uropathogenic Escherichia coli. Science. 1999;285(5430):1061–6. 10446051 10.1126/science.285.5430.1061

[23] Oganesyan V, Gao C, Shirinian L, Wu H, Dall'Acqua WF. Structural characterization of a human Fc fragment engineered for lack of effector functions. Acta crystallographica Section D, Biological crystallography. 2008;64(Pt 6):700–4. PubMed Central PMCID: PMC2467532. 10.1107/S0907444908007877 18560159 PMC2467532

[24] Nowakowski A, Wang C, Powers DB, Amersdorfer P, Smith TJ, Montgomery VA, et al Potent neutralization of botulinum neurotoxin by recombinant oligoclonal antibody. Proceedings of the National Academy of Sciences of the United States of America. 2002;99(17):11346–50. PubMed Central PMCID: PMC123259. 10.1073/pnas.172229899 12177434 PMC123259

[25] Volk WA, Bizzini B, Snyder RM, Bernhard E, Wagner RR. Neutralization of tetanus toxin by distinct monoclonal antibodies binding to multiple epitopes on the toxin molecule. Infect Immun. 1984;45(3):604–9. PubMed Central PMCID: PMC263337. 6205994 10.1128/iai.45.3.604-609.1984 PMC263337

[26] Zwick MB, Wang M, Poignard P, Stiegler G, Katinger H, Burton DR, et al Neutralization synergy of human immunodeficiency virus type 1 primary isolates by cocktails of broadly neutralizing antibodies. Journal of virology. 2001;75(24):12198–208. PubMed Central PMCID: PMC116117. 10.1128/JVI.75.24.12198-12208.2001 11711611 PMC116117

[27] Piccoli L, Campo I, Fregni CS, Rodriguez BM, Minola A, Sallusto F, et al Neutralization and clearance of GM-CSF by autoantibodies in pulmonary alveolar proteinosis. Nature communications. 2015;6:7375 PubMed Central PMCID: PMC4477037. 10.1038/ncomms8375 26077231 PMC4477037

[28] DiGiandomenico A, Keller AE, Gao C, Rainey GJ, Warrener P, Camara MM, et al A multifunctional bispecific antibody protects against Pseudomonas aeruginosa. Sci Transl Med. 2014;6(262):262ra155 10.1126/scitranslmed.3009655 25391481

